# Identification of 2,3,7,8-tetrachlorodibenzo-p-dioxin (TCDD)-inducible genes in human amniotic epithelial cells

**DOI:** 10.1186/1477-7827-4-27

**Published:** 2006-05-17

**Authors:** Yumiko Abe, Hiromitsu Sinozaki, Takeshi Takagi, Takashi Minegishi, Koichi Kokame, Kenji Kangawa, Miki Uesaka, Kaoru Miyamoto

**Affiliations:** 1 Department of Gynecology and Reproductive Medicine, Gunma University Graduate School of Medicine, Maebashi, Gunma, Japan; 2 Education and Research Center of Graduate School of Medicine, Gunma University Graduate School of Medicine, Maebashi, Gunma, Japan; 3 National Cardiovascular Center Research Institute, Osaka, Japan; 4 Department of Biochemistry, Faculty of Medical Sciences, University of Fukui, Fukui, Japan

## Abstract

**Background:**

Exposure to dioxins results in a broad range of pathophysiological disorders in human fetuses. In order to evaluate the effects of dioxins on the feto-placental tissues, we analyzed the gene expression in 2,3,7,8-tetrachlorodibenzo-p-dioxin (TCDD) treated primary cultures of human amniotic epithelial cells.

**Methods:**

Human amniotic epithelial cells were dispersed by trypsin from amniotic membranes and cultured in DME/Ham's F12 medium supplemented with 10% FBS. Two weeks after plating, cells were treated with 50 nM TCDD or DMSO (control), further incubated for 48 hrs, and the gene expression was analyzed by DNA microarray technology and quantitative real-time PCR.

**Results:**

Thirty eight TCDD-inducible genes, including cytochromeP4501A1 and cytochromeP4501B1, were identified. One of the remarkable profiles of the gene expression was the prominent up-regulation of interferon-inducible genes. The genes involved in the interferon gene expression and interferon signaling pathways were also up-regulated. Furthermore, the expression of genes related to collagen synthesis or degradation was enhanced by TCDD.

**Conclusion:**

Using DNA microarray and quantitative real-time PCR analyses, we identified TCDD-inducible genes, including interferon-inducible genes and genes related to collagen synthesis or degradation, in human amniotic epithelial cells.

## Background

Exposure to dioxins causes a diverse spectrum of toxicities in humans and laboratory animals [1-4]. The fetus is one of the most sensitive targets of dioxins and a broad range of pathophysiological abnormalities, such as, disorders of brain development, thyroxin resistance, hepatic damage, hematopoietic disorders and lung dysfunction, are observed in humans after perinatal exposure to dioxins [1]. Dioxins are transferred to fetuses and infants through placentas and milk from mothers [5]. Dioxins were detected in all of the samples analyzed in a study performed using human umbilical cord or cord serum in Japan [6]. Higher dioxin levels were reported in the placenta compared to that in breast milk, in a study performed in Taiwan [7]. Not only the morphological and functional disorders brought about by the altered gene products but the comprehensive analyses of the change in gene expression are required to evaluate the effects of fetal exposure to endocrine disruptors [8]. However those studies of 2,3,7,8-tetrachlorodibenzo-*p*-dioxin (TCDD) on human feto-placentas are limited [6-13].

The amniotic membrane is of fetal origin but contains no blood vessels [14]. The amniotic epithelial cells (AEC) on the basement membrane which is the thickest among basement membranes in human lay in the most interior of the fetal membranes. From the histological character mentioned above, it is relatively easier to obtain homogenous cells for experiments without contamination of hematocytes or cells of blood vessel origin [15]. Moreover, since fetal membranes are the tissues disposed of after parturition, the ethical issues associated with use for experiments are limited. Therefore, we analyzed gene expression induced by TCDD, the most toxic congener among the dioxins, in primary cultures of human AEC using DNA microarray technology, a powerful tool in revealing global gene expression, and quantitative real-time PCR.

## Methods

### Reagents

2,3,7,8-TCDD was obtained from Cambridge Isotope Laboratory (Andover, MA). Human oligonucleotide glass array (Human Genome U133A Array) was from Affymetrix (Santa Clara, CA).

### AEC culture and TCDD treatment

With the permission of The Internal Review Board for Gunma University Hospital and the written informed consent of the patients, we obtained fetal membrane samples from patients with single pregnancy without systemic disease, signs of premature delivery or fetal complications, during elective cesarean sections at term. Three fetal membranes from three women were used for independent primary cultures. AEC were prepared based on the method described by Okita et al [15] with slight modifications, after an amniotic membrane was mechanically peeled off from the chorion. Briefly, the membrane was incubated in 170 ml of Krebs-Ringer solution containing 0.15 % trypsin, 1.26 g/l sodium bicarbonate, 25 mM HEPES, 50 μg/ml streptomycin and 0.25 μg/ml amphotericin B at 37C using a Spinner Flask. The liberated cells were decanted at 30-min intervals and the incubation was performed seven times with freshly made trypsin solution. Each fraction of dispersed cells were centrifuged, resuspended in DME/Ham's F12 medium supplemented with 15 mM HEPES, 10% FBS, 50 μg/ml streptomycin and 0.25 μg/ml amphotericin B. The first fraction was discarded. The cell viability of the remaining fractions was determined by trypan blue exclusion and the fractions with viabilities not less than 80% were pooled. Cells were plated at the density of 2 × 10^6 ^cells per 25 cm^2 ^polystyrene flask in 10 ml of medium and cultured in a humidified atmosphere containing 5% CO_2 _– 95% air at 37C. The medium was changed every 2 to 3 days. The experiments were performed two weeks after plating when cells were sub-confluent to confluent. The cells were treated with 50 nM TCDD or DMSO (control), and further incubated for 48 hrs. Total RNA from these cells was isolated and used for DNA microarray and quantitative Real-Time PCR analyses.

### DNA microarray

The microarray method was carried out according to the manufacture's instruction. Briefly, total RNA was extracted from the TCDD-treated cells and control AEC using an RNA extraction solution (Trizol). After Oligo-dT latex beads treatment, double stranded cDNA libraries were constructed from the mRNA of TCDD-treated cells and the control AEC using an oligo-dT primer with a T7-promoter sequence at the 5'-end. Biotin-labeled complementary RNA was in vitro transcribed by T7 promoter using the cDNA libraries as template. The biotin-labeled RNA was fragmented. Each sample was hybridized to a separate oligonucleotide array (Affymetrix Human Genome U133A) for 16 hours at 45C, and then washed and stained with streptavidin phycoerythrin conjugate using GeneChip Fluidics. The arrays were scanned by a Gene-Array scanner using Affymetrix GeneChip Microarray Suite (MAS) 5.0 software for scanning and basic analysis.

The Human Genome U133A array contains 22,277 probe sets including 61 control probe sets, and analyzes the expression level of 18,720 full length transcripts with 13,900 characterized human genes. The arrays incorporate a perfect match (PM)/mismatch (MM) probe pair strategy. One probe from the pair perfectly matches its target sequence while the other contains a mismatch located at the center of the 25-mer sequence. This probe pairing design helps identify and subtract non-specific hybridization and background signal. For each probe pair, the MM signal was subtracted from the PM signal. The average of these differences was reflective of the level of expression of the gene. The global method of scaling/normalization was performed by MAS 5.0 software. A program (PathwayFinder) was used for pathway analysis. Three independent experiments were performed.

### Quantitative real-time PCR

Messenger RNA was extracted using an RNA extraction solution (Trizol) and oligo-dT latex beads as described previously [15,16]. Five micrograms of mRNA preparations were reverse-transcribed, and then converted to double stranded cDNA molecules. Complementary DNA was quantified by UV absorption measurement, and 1 ng was subjected to the PCR reaction as template. As an internal standard, TATA binding protein (TBP) was used instead of GAPDH, since GAPDH gene expression was affected by the TCDD treatment (data not shown). Quantitative real-time PCR was carried out in an ABI PRISM^® ^7000 Sequence Detection System (Applied Biosystems; Foster City, CA), according to the manufacture's instructions. PCR reaction involved template cDNA samples, Advantage Taq Plus DNA polymerase (Clontech Laboratories, Inc.; Mountain View, CA), dNTP, and Syber Green. The thermal cycling conditions included an initial incubation of samples at 94°C for 2 min, followed by appropriate cycles of 94°C for 20 sec, 54°C for 30 sec and 72°C for 45 sec. Syber Green fluorescence was used to detect the amplified products. Serial dilutions of the templates were used to create a concentration curve, and relative expression levels were calculated using TBP as normalization control for each sample. Abundance of each gene was referred to as a Ct value in this system [16,17]. Three independent experiments using AEC from three women were performed. Comparisons between the genes were performed using one-way ANOVA. The significance of the differences between the mean values of cytochromeP4501A1 (CYP1A1) and each gene was tested using paired *t*-test. P < 0.05 was considered statistically significant.

## Results

Among the 22,277 genes spotted on the array, 12,509 genes were expressed in AEC population. Six hundred and ninety six TCDD-sensitive candidate genes were picked up; 326 were TCDD-inducible and 370 were TCDD-suppressive candidate genes (cut-off values of 1.9 as inducible and 0.5 as suppressive genes). Specific primers for 55 genes among 696 TCDD-sensitive candidate genes were synthesized (Table 1). Those genes were selected for two reasons. One reason was that TCDD-sensitive genes in rat placenta had been examined previously [16]. In order to reveal the relationship of sensitive genes between rat placenta and AEC, those genes were selected. Another reason was that even the candidate genes might not represent actual sensitive genes, because false positive signals could not be excluded from the array data, especially when induction or suppression ratios were near 1.9 or 0.5. Therefore we selected genes with higher ratios for real-time PCR.

**Figure 1:**
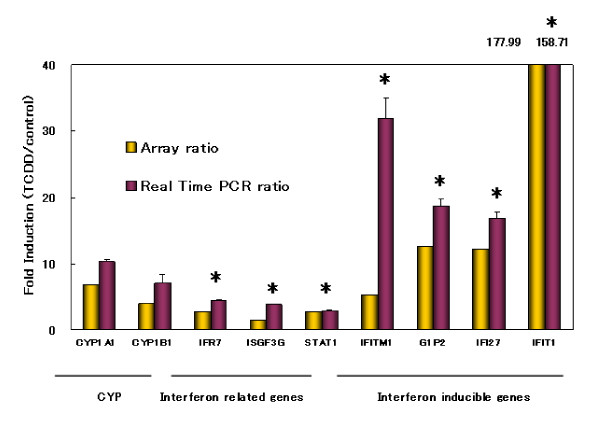
Induction of CYP1A1, CYP1B1 and interferon related genes in human AEC by TCDD. Expression levels were determined by DNA microarray and quantitative real-time PCR analyses as described in *Methods*. Values were expressed as fold induction (TCDD/control). The representative microarray data are shown as yellow columns. The values obtained by PCR analyses are shown as red columns. The data are shown as mean ± SD of three independent experiments. *, P < 0.05 compared with the value of CYP1A1.

Genes that showed expression ratios (TCDD-treated/control) of more than 1.9 or less than 0.6 were finally identified as TCDD-sensitive. Among 55 genes analyzed, 38 genes were TCDD-inducible (Table 2), 4 were -suppressive (Table 3), and 13 were neither of them, by both DNA microarray technology and quantitative real-time PCR. TCDD-inducible genes were categorized into several groups. Enzyme genes include CYP1A1 and cytochromeP4501B1 (CYP1B1) which are known as the typical TCDD target genes. One of the remarkable findings of the present experiment was that many interferon-related genes were induced in TCDD-treated human AEC (Figure 1). The expression of interferon-inducible genes, that is, interferon induced transmembrane protein 1 (IFITM1), interferon, alpha-inducible protein (G1P2), interferon, alpha-inducible protein 27 (IFI27) and interferon-induced protein with tetratricopeptide repeats 1 (IFIT1) was strongly enhanced. The genes involved in the interferon gene expression and signaling pathway, that is, interferon regulatory factor 7 (IFR7), interferon-stimulated transcription factor 3, gamma 48 kDa (ISGF3G) and signal transducer and activator of transcription 1 (STAT1), were also up-regulated.

**Table 1:** Primers used for quantitative real-time PCR

Gene Name	Abbreviation		Primers
Arylsulfatase B	ARSB	Forward Primer	GTGGTGTGATCTCGGCTCACT
		Reverse Primer	CGTGGTGGTGTATGCCTGTAAT
Carbohydrate (N-acetylglucosamine 6-O) sulfotransferase 6	CHST6	Forward Primer	GAAACTGAGTCCACCACTTGAGAA
		Reverse Primer	TTATTGCTCCTAAAGTCTTCGACTTG
Cytochrome P450 1A1	CYP1A1	Forward Primer	AGCGGAAGTGTATCGGTGAGA
		Reverse Primer	CTGAATTCCACCCGTTGCA
Cytochrome P450 1B1	CYP1B1	Forward Primer	AGCAGGCTTGCCCAGTACATT
		Reverse Primer	AAATAGGCTACAGCAGCCCAAA
Dehydrogenase/reductase (SDR family) member 2	DHRS2	Forward Primer	TACTCATGCTAGGCTTGAGGAAGA
		Reverse Primer	CACCAAGAACCCCACATGTTAA
Fucosyltransferase 2 (secretor status included)	FUT2	Forward Primer	CCAGTGTGCATACAGTCATGGA
		Reverse Primer	CACGGTGCACTATATTCCCTAACTC
GDP-mannose 4,6-dehydratase	GMDS	Forward Primer	GCCAAGGACTATGTGGAGGCTAT
		Reverse Primer	ACAAATTCCCGGACACTATGGA
Matrix metalloproteinase 9	MMP9	Forward Primer	TTCCAGTACCGAGAGAAAGCCTAT
		Reverse Primer	GGTCACGTAGCCCACTTGGT
Membrane metallo-endopeptidase	MME	Forward Primer	ATTCCTTTGGGCCTCTGCTT
		Reverse Primer	TGGGAAGGCAGCATTGGA
Mitochondrial thioredoxin reductase	TXNRD2	Forward Primer	CTGAGGAAACTCTTATCAGAACATTACAC
		Reverse Primer	GCGACGCGGTGCTACAA
Podocalyxin-like	PODXL	Forward Primer	GCAGAGAGGGCAAGAGTAAAACTG
		Reverse Primer	GAGTCATCTGTGTCCTCCATGCT
Poly (ADP-ribose) polymerase family, member 12	ZC3HDC1	Forward Primer	TTCTCAGAGTCTCATGGCATCATAGT
		Reverse Primer	GTCAGAACAACAGGCAGAAGTGA
Stearoyl-CoA desaturase	SCD	Forward Primer	AGGAATGTCCACCATGAACTTGATA
		Reverse Primer	CACCGCTTCTCCAATGGATT
Ubiquitin-conjugating enzyme E2E 1 (UBC4/5 homolog, yeast)	UBE2E1	Forward Primer	GGTGGGAAGTATTGCCACTCA
		Reverse Primer	GTGAAACCCCAATTTATGTAGCGTAT
Uridine phosphorylase 1	UPP1	Forward Primer	CGATTAAGAGACAGAGAATCTTGGATTA
		Reverse Primer	GAAACCCCAAATCAGGCTAACA
Interleukin 12B	IL12B	Forward Primer	GACAAGTAGTTATGGCTAAGGACATGA
		Reverse Primer	AGGGATTCCAGATTTTCTTTGCA
Distal-less homeo box 2	DLX2	Forward Primer	AGCCTGGACTTGGACACAGAGT
		Reverse Primer	GGGTTGCTGAGGTCACTGCTA
Early growth response 1	EGR1	Forward Primer	AAGCCAAGCAAACCAATGGT
		Reverse Primer	ACTCTGACACATGCTCTGAGAATACTG
High mobility group AT-hook 1	HMGA1	Forward Primer	GTCCCCTACTCCCTCTTCACTGT
		Reverse Primer	ACCTGGACAATAAGTGACTGCATCT
Interferon regulatory factor 7	IFR7	Forward Primer	GCCTGGTCCTGGTGAAGCT
		Reverse Primer	GAAGCACTCGATGTCGTCATAGA
Interferon-stimulated transcription factor 3, gamma 48 kDa	ISGF3G	Forward Primer	AAGTAGACTCATTCTTCACACGATTGAC
		Reverse Primer	AGCCAGTGTGTGCGAGGATT
Myeloid/lymphoid or mixed-lineage leukemia (trithorax homolog, Drosophila); translocated to, 10 (MLLT10)	MLLT10	Forward Primer	GACCATTAAAAGCTCACCACTAGAGTTC
		Reverse Primer	CTGCGGTACTGTCACATCAAAAG
N-myc (and STAT) interactor (NMI)	NMI	Forward Primer	CGTGAAGATCAAATGAGAGACAAACT
		Reverse Primer	CTCCCGGACTGTCTGTCATAGTC
Nuclear antigen Sp100	SP100	Forward Primer	CAGCTGTTTTGTTGACATTCTGAA
		Reverse Primer	TGGAAGAAGACTGACCTGGTACCT
Tumor necrosis factor, alpha-induced protein 3	TNFAIP3	Forward Primer	GAGTAAATTGGCCTCTTTGATACACTT
		Reverse Primer	AGGAGAAGCACGAAACATCGAT
v-maf musculoaponeurotic fibrosarcoma oncogene homolog F (avian)	MAFF	Forward Primer	CCAGAAGGCAGAGGTTGTAGTGA
		Reverse Primer	AGGAGCCGGGCAATATTTTTTA
GABA-B receptor	GPR51	Forward Primer	TGTGCGTCTGTAACCCTTTGTG
		Reverse Primer	AATGGGTGAACCTACAGTATCAGTAAGA
Interferon, alpha 1	IFNA1	Forward Primer	TGATGAATGCGGACTCCATCT
		Reverse Primer	GACAACCTCCCAGGCACAAG
Signal transducer and activator of transcription 1	STAT1	Forward Primer	TTGAGTGGATGATGTTTCGTGAA
		Reverse Primer	AGAACCTTGTCAAACCCATCTCTT
Solute carrier family 2 (facilitated glucose transporter), member 1	SLC2A1	Forward Primer	ACCACTGCAACGGCTTAGACTT
		Reverse Primer	TGGGTAACAGGGATCAAACAGAT
Solute carrier family 2 (facilitated glucose transporter), member 3	SLC2A3	Forward Primer	TTGCTCTGGGTGGAAGTACGTT
		Reverse Primer	ACCAAGAAGGGAAAGGGAGACT
Amphiregulin	AREG	Forward Primer	GCTGCCTTTATGTCTGCTGTGA
		Reverse Primer	CGTTCCTCAGCTTCTCCTTCA
Caveolin 2	CAV2	Forward Primer	CTCATATCCTTTTGAAGGTAGTTGCA
		Reverse Primer	GGTGATGCTTTAAGGTAATGATTATGC
DnaJ (Hsp40) homolog, subfamily C, member 3	DNALC3	Forward Primer	AAGAAGTTTGACGACGGAGAAGA
		Reverse Primer	TTGAACCCTTGCCATGAGTTC
Epiregulin	EREG	Forward Primer	CAATGTAACTCCACTGTTCTCCTGAA
		Reverse Primer	CTGGTGGTGATTGAATTTAGTCTCA
Epithelial membrane protein 1	EMP1	Forward Primer	AACTCTTGTGGTACCTAGTCAGATGGTA
		Reverse Primer	GCAAAGCAATGCCTGCTTAAC
FLJ20035	FLJ20035	Forward Primer	TAGTCCAGGATAACAGGATGAATGAA
		Reverse Primer	ACATAGCTCACGCAAGGAAACA
Insulin induced gene 1	INSIG1	Forward Primer	AAGCTTAGAGGAACTTGCCTGTGA
		Reverse Primer	TACTCCAAGACATTTCCCTCAAAAC
Integrin, alpha 10	ITGA10	Forward Primer	AGTAAAGGCAGTTGGATTCTCATAGAC
		Reverse Primer	GAGCTGCACTCTGGAGACCAAT
Integrin, alpha 2 (CD49B, alpha 2 subunit of VLA-2 receptor)	ITGA2	Forward Primer	AGATGATTTGGTCAGATTGGGATAAG
		Reverse Primer	TGGGTGGTGTTTCTCAAAGTGT
Interferon induced transmembrane protein 1	IFITM1	Forward Primer	TCCCTGTTCAACACCCTCTTCT
		Reverse Primer	GTCACGTCGCCAACCATCTT
Interferon, alpha-inducible protein	G1P2	Forward Primer	CCTGCTGGTGGTGGACAAAT
		Reverse Primer	CCGCTCACTTGCTGCTTCA
Interferon, alpha-inducible protein 27	IFI27	Forward Primer	TGGCCAGGATTGCTACAGTTG
		Reverse Primer	TATGGAGGACGAGGCGATTC
Interferon-induced protein with tetratricopeptide repeats 1	IFIT1	Forward Primer	GAAACTTCGGAGAAAGGCATTAGA
		Reverse Primer	GCTCATAGTACTCCAGGGCTTCAT
Interferon-induced protein with tetratricopeptide repeats 2	IFIT2	Forward Primer	CTTGGAACGATTGAGATTTTCTAGGT
		Reverse Primer	CCCAGAGTGTGGCTGATGCT
Keratin 6A	KRT6A	Forward Primer	TTCAGAACAACTTCCACTTACTTTCC
		Reverse Primer	GTCACTTGTGCTTTCATGGATACTG
Low density lipoprotein receptor-related protein 3	LRP3	Forward Primer	CCCATCCTATGGTCAGCTCATC
		Reverse Primer	CGTGCCGACGCATCTGT
Presenilin 2 (Alzheimer disease 4)	PSEN2	Forward Primer	CACAGCAGGTTTATCCAGATGAAC
		Reverse Primer	CACTCCCGAGCACACTCTTTG
Ras-related associated with diabetes	RRAD	Forward Primer	TTGAGACATCAGCGGCATTG
		Reverse Primer	CGTCGTGCGTTGGCTTCT
Serine (or cysteine) proteinase inhibitor, clade B (ovalbumin), member 2	SERPINB2	Forward Primer	GTGAATGAGGAGGGCACTGAAG
		Reverse Primer	AGAAAAGGATGATCTGCCACAAAC
Small proline-rich protein 3	SPRR3	Forward Primer	CCATGTCCTTCAACGGTCACT
		Reverse Primer	AGCATCTGGTGGTTGGCTTCT
Tripartite motif-containing 14	TRIM14	Forward Primer	GCAGAGACAGAGCTAGACTGTAAAGGT
		Reverse Primer	CCTGGTCACACAATTGATATGGA
Tumor necrosis factor	TNF	Forward Primer	GAATGTGTGGCCTGCACAGT
		Reverse Primer	CCAGATGTCAGGGATCAAAGC
Chromosome 10 open reading frame 116	C10orf116	Forward Primer	ACAGCCTGGCCCTGATCTC
		Reverse Primer	GCTTGCGAGGAATCATGAAGT
Pleckstrin homology-like domain, family A, member 1	PHLDA1	Forward Primer	ACGAGCACATTTCTATTGTCTTCACT
		Reverse Primer	TCGCAAGTTTTCAGTAGGGTGAT

**Table 2:** TCDD-inducible genes in human AEC

Molecular function	Gene Name	Array	Real Time	Ct	Accession
		ratio	PCR ratio		
Enzyme	Carbohydrate (N-acetylglucosamine 6-O) sulfotransferase 6	2.46	1.92	33.82	NM_021615
	Cytochrome P450 1A1	6.79	10.36	20.74	NM_000499
	Cytochrome P450 1B1	3.99	7.13	19.36	NM_000104
	Dehydrogenase/reductase (SDR family) member 2	2.57	3.17	26.62	NM_182908
	Fucosyltransferase 2 (secretor status included)	2.85	1.99	27.64	NM_000511
	Matrix metalloproteinase 9	4.49	4.67	23.58	NM_004994
	Membrane metallo-endopeptidase	2.75	2.88	27.60	NM_007287
	Podocalyxin-like	3.50	3.83	22.26	NM_005397
	Poly (ADP-ribose) polymerase family, member 12	4.02	4.57	26.69	NM_022750
	Stearoyl-CoA desaturase	2.56	2.06	27.95	AB032261
	Uridine phosphorylase 1	2.56	2.40	31.76	NM_003364
Transcription Factor	Distal-less homeo box 2	3.05	2.50	24.50	NM_004405
	Interferon regulatory factor 7	2.82	4.46	25.21	NM_004030
	Interferon-stimulated transcription factor 3, gamma 48 kDa	1.54	3.78	25.44	NM_006084
	N-myc (and STAT) interactor (NMI)	2.61	3.30	26.18	NM_004688
	Nuclear antigen Sp100	1.06	2.28	29.04	NM_003113
	Tumor necrosis factor, alpha-induced protein 3	2.50	2.18	25.94	NM_006290
	v-maf musculoaponeurotic fibrosarcoma oncogene homolog F (avian)	3.33	3.75	27.11	NM_012323
Signal Transducer	GABA-B receptor	2.59	2.56	29.53	AF056085
	Signal transducer and activator of transcription 1	2.84	2.85	21.55	NM_007315
Structural Protein and Other Groups	Amphiregulin	2.80	10.70	22.56	NM_001657
	Epiregulin	4.18	3.19	27.35	NM_001432
	Epithelial membrane protein 1	3.01	2.84	25.54	NM_001423
	FLJ20035	11.16	5.58	30.53	NM_017631
	Insulin induced gene 1	2.53	2.61	26.85	NM_005542
	Integrin, alpha 10	2.72	1.91	25.04	AF112345
	Integrin, alpha 2 (CD49B, alpha 2 subunit of VLA-2 receptor)	2.42	3.54	25.17	NM_002203
	Interferon induced transmembrane protein 1	5.31	31.85	27.24	NM_003641
	Interferon, alpha-inducible protein	12.65	18.72	21.80	NM_005101
	Interferon, alpha-inducible protein 27	12.13	16.84	23.13	NM_005532
	Interferon-induced protein with tetratricopeptide repeats 1	177.99	158.71	26.34	NM_001001887
	Keratin 6A	3.23	4.45	21.99	NM_005554
	Ras-related associated with diabetes	2.72	2.42	23.57	NM_004165
	Serine (or cysteine) proteinase inhibitor, clade B (ovalbumin), member 2	11.00	12.36	24.86	NM_002575
	Small proline-rich protein 3	2.97	3.56	22.33	NM_005416
	Tripartite motif-containing 14	4.61	2.56	27.43	NM_014788
	Tumor necrosis factor	3.89	3.04	26.79	NM_000594
Function Unknown	Pleckstrin homology-like domain, family A, member 1	2.81	5.13	27.91	NM_007350

**Table 3:** TCDD-suppressive genes in human AEC

Molecular function	Gene Name	Array ratio	Real Time PCR ratio	Ct	Accession
Signal Transducer	Interferon, alpha 1	5.03	0.22	34.80	NM_024013
Transporter	Solute carrier family 2 (facilitated glucose transporter), member 3	0.54	0.38	23.13	NM_006931
Structural Protein	Low density lipoprotein receptor-related protein 3	2.69	0.56	26.61	NM_002333
Function Unknown	Chromosome 10 open reading frame 116	2.74	0.28	33.78	NM_006829

The other aspect in TCDD-treated human AEC was that integrin, alpha 10 (ITGA10), integrin, alpha 2 (ITGA2), and matrix metalloproteinase 9 (MMP9), which were genes involved in collagen synthesis and degradation, were also induced by TCDD treatment. However no changes in cellular morphology, number and viability were observed under the experimental conditions.

## Discussion

The amniotic membrane composes the innermost layer, nearest the fetus, of the fetal membranes containing the fetus and amniotic fluid [14]. It contains no blood vessels or nerves; the nutrients it requires are supplied by the amniotic fluid. The amniotic epithelium derived from epiblasts of embryo, laid in the most interior of the amniotic membrane, is in contact with amniotic fluid directly. The DNA microarray is a powerful and comprehensive method to identify inducible or suppressive genes by a given hormonal or pharmacological treatment. Using DNA microarray and quantitative real-time PCR technology, we analyzed TCDD-inducible genes in primary cultures of human AEC.

We measured gene expression after 48 hour incubation. Therefore early induced genes are not included in the present study. Biological persistence of TCDD is well known. It is reported that the expression of CYP1A1 mRNA was shown after 6 hours and sustained for up to 72 hours in human colon carcinoma cells treated with TCDD [18]. The maximum effect of TCDD on the expression of FSH receptor mRNA in rat granulosa cell cultures was shown after a 48 hour-incubation in our previous study [19]. The effect of TCDD on the expression of LH receptor mRNA was shown after 24 hours and sustained for up to 72 hours in the same cells [20]. Therefore, we studied TCDD-inducible genes in human AEC after a 48 hour-incubation.

The most striking observation in this experiment was that many interferon-inducible genes were prominently up-regulated in TCDD-treated human AEC; the increase of gene expression of IFITM1, G1P2, IFI27 and IFIT1 was equal to or far more than that of CYP1A1 and CYP1B1 which are well known as TCDD-inducible genes. The genes involved in the interferon gene expression and signaling pathway, that is, IFR7, ISGF3G and STAT1, were also up-regulated. The induction of interferon-related genes by TCDD was first reported by Mizutani et al., in the placentas of TCDD-treated Holtzman rats [16]. The present study confirmed that TCDD induces interferon-related genes in cells derived from human fetuses.

On the other hand, the up-regulation of glucose transporter genes was not observed in AEC culture by DNA microarray analysis (data are not shown), though they were strongly up-regulated in TCDD-treated rat placentas [16]. The placenta is the organ rich in blood vessels and it is reported that exposure to TCDD causes a hypoxic state in the placentas and the glucose kinetics are also altered in those organs [21,22]. On the other hand, interferon is known to be involved in the regulation of angiogenesis. Mizutani et al speculated that the activation of the interferon signaling pathway impaired the angiogenesis in TCDD-treated rat placentas and brought about a hypoxic state in the placentas which up-regulated glucose transporter gene expression [16]. It was deduced that the expression of glucose transporter genes was not up-regulated in AEC, since both the control and TCDD-treated cells had an adequate supply of oxygen when in the *in vitro *culture condition. AEC cultures in the present study were performed under air conditions. In future studies, comparing TCDD-inducible genes between AEC cultures under hypoxic conditions and under air conditions would verify the inference and show which condition is closer to *in vivo*.

Although no changes in cellular morphology, number and viability were observed under the experimental conditions, it was an interesting observation that the expression of genes related to the synthesis and degradation of collagen, that is, MMP9, ITGA2 and ITGA10 were also up-regulated by TCDD in human AEC. Martinez JM et al reported the induction of MMP1, MMM9 and tissue inhibitor of metalloproteinase 3 in TCDD-treated human airway epithelial cells using microarray analysis in 2002 [23]. Thereafter, increased mRNA levels of MMPs were reported in TCDD-treated human cells, such as, MMP1 in keratinocytes [24] and melanoma cells [25], MMP2 in melanoma cells [25], MMP3 and MMP7 in endometrial cells [26], and MMP9 in prostate cancer cells [27] and melanoma cells [25]. Although the mechanisms of induction of MMP genes by TCDD are not fully clarified, Murphy et al reported that TCDD activation of MMP1 mRNA expression was mediated through increased promoter activity [24]. Villano CM et al reported aryl hydrocarbon receptor dependent MMP1, 2, and 9 expression by TCDD and speculated that MMP expression may be a common endpoint for activation of the aryl hydrocarbon receptor pathway [25]. It is speculated that TCDD caused pathological lesions by altering the expression of genes involved in matrix remodeling [24]. On the other hand, increased premature labor is reported in women exposed to dioxins in Chapaevsk [28]. The tensile strength of fetal membranes is provided almost exclusively by the amnion, and the interstitial collagens are believed to be the major source of the tensile strength of this tissue. AEC secrete not only collagens and noncollagenous glycoproteins but also MMPs [14]. Considering these biological characters of the amnion and the present observations on increased expression of genes related to the synthesis and degradation of collagens, our findings permit a better understanding of the pathology of premature labor by dioxins. The study of mechanisms on the induction of these genes by TCDD in human AEC would be the subject of future work.

The data of TCDD-inducible and -suppressive genes in human AEC culture is available at ED-Genes [29].

## Conclusion

We identified 38 TCDD-inducible genes in human AEC using DNA microarray and quantitative real-time PCR analyses. Interferon-inducible genes and genes related to collagen synthesis or degradation were up-regulated. These observations indicate that the comprehensive methods used in this study are useful in investigating the effects of dioxins on human feto-placenta.
